# Self-Disclosure and Non-Communication: Stigma Management in Third-Sector Transitional Employment

**DOI:** 10.3390/ijerph182211840

**Published:** 2021-11-11

**Authors:** Miira Niska, Melisa Stevanovic, Elina Weiste, Tommi Ostrovskij, Taina Valkeapää, Camilla Lindholm

**Affiliations:** 1 Faculty of Social Sciences, University of Helsinki, 00014 Helsinki, Finland; tommi.ostrovskij@helsinki.fi (T.O.); taina.valkeapaa@helsinki.fi (T.V.); 2 Faculty of Social Sciences, Tampere University, 33100 Tampere, Finland; melisa.stevanovic@tuni.fi; 3 Finnish, Finno-Ugrian and Scandinavian Studies, University of Helsinki, 00014 Helsinki, Finland; Elina.Weiste@ttl.fi; 4 Finnish Institute of Occupational Health, 00250 Helsinki, Finland; 5 The Languages Unit, Tampere University, 33100 Tampere, Finland; camilla.lindholm@tuni.fi

**Keywords:** stigma, mental illness, employment, stigma management, rehabilitation, self-disclosure, non-communication, discursive social psychology

## Abstract

People who are recovering from a mental illness often have difficulties finding and maintaining employment. One of the main reasons for these difficulties is the negative label, or stigma, attached to mental illnesses. People who possess stigmatizing characteristics may use compensatory stigma management strategies to reduce discrimination. Due to mental illnesses’ invisible characteristics, information control is an important stigma management strategy. People can often choose whether they disclose or non-communicate their illness. Nevertheless, it might be difficult to decide when and to whom to disclose or non-communicate the stigma. Since stigma management is a dilemmatic process, workers in mental health services play an important role in informing their clients of when it is best to disclose or non-communicate their illness. In this article, we adopt the perspective of discursive social psychology to investigate how workers of one mental health service programme evaluate and construct self-disclosure and non-communication as stigma management strategies. We demonstrate how these workers recommend non-communication and formulate strict stipulations for self-disclosure. At the same time, they differentiate non-communication from lying or providing false information. The study contributes to an improved understanding of stigma management in contemporary mental health services.

## 1. Introduction

The employment of people who are recovering from a mental illness is a major societal aim across the world. The participation of rehabilitees in meaningful work is beneficial for them and is necessary for societies that struggle with the economic burden caused by mental disorders [1]. At the same time, people who are recovering from a mental illness have great difficulties finding and maintaining employment [2]. The key problem is the negative label attached to mental illnesses. This label is commonly conceptualised as stigma.

Following the classic formulation of Goffman [3], stigma is a characteristic that ‘discredits’ a person, reduces social standing and leads to problematic relations between the ‘normal’ and the ‘stigmatized’. Stigma may be conceptualized as a public, experienced, perceived, or internalized phenomenon [4,5] and it is commonly discussed as something inflicted upon the stigmatized from the outside or as something that a person is able to influence [6]. Another type of stigma is an affiliate stigma [7], or a courtesy stigma [3], which indicates that stigmas also affect those who are closely associated with stigmatized individuals.

In the contemporary world, mental illness is among the most stigmatizing characteristics a person can possess. People with mental illnesses are portrayed as dangerous, unpredictable and violent [8,9]. Such negative stereotypes typically lead to discrimination in, for example, housing and employment [10]. Stigmatizing attitudes toward people with mental illnesses have also been observed across the healthcare service industry [11,12], although specific training in mental health has improved attitudes toward people with mental illnesses [13].

Following Goffman [3], researchers have become interested in the process of stigma management [14,15,16]. Stigmas are constructed during interactions between stigmatized and non-stigmatized individuals. Therefore, a stigmatized person has an opportunity to affect others’ perceptions and reactions to their stigma [15]. According to the Stigma Management Communication Model, a person can manage their stigma by accepting, denying, avoiding, ignoring/displaying, evading responsibility, and reducing offensiveness [15]. The effectiveness of these strategies may vary in different stigmatizing situations and in the wrong context, can even be damaging [17].

Although mental illness is a stigmatizing characteristic, it is not a visible attribute in the same way that skin colour is, for example. People with mental illnesses are often able to conceal their stigma if they choose to do so [10]. In the case of concealable stigmas, an important stigma management strategy consists of information control; people can choose when and to whom they disclose their stigma [3]. In other words, people with mental illness often have the choice of either disclosing or non-communicating their illness. Nevertheless, it might be difficult to choose when and to whom to do, or not do, this. Disclosure may lead to discrimination, but non-communication may lead to accusations of lying and deception.

Since information control is a dilemmatic stigma management strategy, mental health service workers play an important role in informing their clients when is best to disclose or non-communicate their illness. Yet, few studies have examined how workers in mental health services work with their clients to manage mental illness stigma [8]. In this study, we adopted the perspective of discursive social psychology [18,19,20,21,22] to investigate the ways in which mental health service workers discuss information control with rehabilitees in the context of Clubhouse-based transitional employment.

Discursive social psychology has been a somewhat underused approach in stigma management studies [23]. Nevertheless, the approach provides a robust way to study stigma management ‘in practice’ [23]. From the perspective of discursive social psychology, rehabilitees and mental health service workers are active discourse users, who situationally construct, negotiate and resist both stigmatized identities [23] and the functional ways through which to manage these identities.

In this study, we investigated how mental health service workers construct self-disclosure and non-communication as stigma management strategies in naturalistic interaction situations instead of, for example, research interviews. The study contributes to improving the understanding of stigma management in contemporary mental health services. Socially constructed ideas of suitable stigma management strategies are part of both mental health service workers’ and rehabilitees’ social reality. An increasing reflexive awareness of the ways in which stigma management strategies can be evaluated and constructed in everyday interaction is important for mental health service workers’ professional development. In addition, such an awareness may aid rehabilitees to reflect on the interaction situations they have participated in, and on the possible discrepancies between their own and other people’s ways of relating to information control in transitional employment.

## 2. Self-Disclosure and Non-Communication in Stigma Management

Self-disclosure refers to the communication of personal information that is not readily available to others [24]. In the contemporary Western world, self-disclosure is a positively evaluated phenomenon. It is commonly connected with trust and positive relationship-building [24,25]. Researchers argue that disclosing secrets, such as information about mental illness, can reduce anxiety [26]. Goffman [3] identified voluntary stigma disclosure as a stigma management strategy. However, stigma disclosure may also lead to discrimination and humiliation [10]. Thus, in some situations, it might be better to non-communicate the stigmatized characteristic than to disclose it.

Non-communication refers to a process in which someone does not communicate something under certain conditions [27]. Although non-communication is often viewed as a failure in communication, it can also be viewed as a natural and important part of communication [28]. Sometimes, communication might alter a situation in a negative way, which means that non-communication may be beneficial for either the non-communicator or some other individual or group. For example, respondents of the Finnish Mental Health Barometer [29] replied that disclosing mental health problems poses a risk to one’s employment. At the same time, non-communication is associated with lying, manipulation and a dubious use of power [25,28]. Thus, in some situations, non-communication may jeopardize trust and social relationships.

Vesala and Knuuttila [28,30] expand on Gregory Bateson’s [27] ideas and identify three aspects of non-communication: contextual embeddedness, systemic functions, and evaluative controversies. First, non-communication takes place and is interpreted in a specific context. In some contexts, it can channel a powerful message. Silence can become an expression of anger if interlocutors are able to create the context and recognize the absence of the message. In other contexts, it might be critical that the very act of non-communication is not communicated to interlocutors [28]. If mental illness is non-communicated in a job interview, it is essential that the recruiter does not recognize the absence of the message.

Second, non-communication commonly occurs because communication in a specific situation would somehow hurt the social system: either the person non-communicating or some other person or group. A person recovering from a mental illness might non-communicate their illness in a job interview in order to protect themselves from the recruiter’s judgement and discrimination. However, such a person might also choose to disclose the mental illness to explain a disjointed career path.

Finally, the evaluation of non-communication is a controversial matter [28]. Non-communication may be viewed as manipulation in one context and praised as a human right in another context. Lying is usually evaluated negatively, but in contrast, white lies, for example, are considered preferable to hurting someone’s feelings. According to Vesala and Knuuttila [28], non-communication is often made meaningful through these types of controversies over its evaluation.

## 3. Materials and Methods

### 3.1. Materials

The data we analysed in this study originate from a project, namely the ’Interaction, social inclusion, and mental illness’ project, that focused on an international, non-governmental organization called the Clubhouse, and their Clubhouse-created Transitional Employment programme. The Clubhouse model involves local community centres that offer people with mental illnesses a place to belong to, in which to interact with others, and to find assistance with regard to employment, education and housing [31]. The Clubhouse is a membership organization, which means it is open to everyone with a history of mental health-related problems. Those who participate in the Clubhouse activities are its members [31]. The role of the paid staff members is to support the Clubhouse members by working side-by-side with them in all functions of the house [31].

The relationship between the Clubhouse members and the staff members is understood as one of equals, involving a commitment to reduce the stigma surrounding mental illnesses [31,32]. This means Clubhouse members are not categorized on the basis of their mental illnesses but are approached as whole persons with individual characteristics. Moreover, anyone who attends the Clubhouse is considered a co-participant with something to contribute to the common issues of the organization. According to the Clubhouse standards, members are not expected to talk about their illnesses unless they specifically wish to do so [31].

One central goal of the Clubhouse model is to advance their members’ re-entry to the labour market [33]. In order to support their members’ work skills and confidence, Clubhouses organise Transitional Employment (TE) training, which is a part-time prevocational training period at an employer’s place of business. The TE workplace is managed by the Clubhouse community so that each member works a TE period from six to nine months. The Clubhouse community, rather than employers or individual members, manages the TE selection, training and replacements [34]. In this article, we analyse discussions that took place in the TE meetings between staff members and Clubhouse members on how to improve contacts between potential employers and the Clubhouse organization.

The study is based on a data set of 26 video-recorded TE meetings from one Finnish Clubhouse. The meetings took place weekly over an 11-month period, from October 2016 to August 2017. In these meetings, stigma management and information control were focal topics. While the participants discussed how to contact potential employers, they also discussed when and to whom they should disclose or non-communicate Clubhouse members’ mental illnesses. Since both staff members and Clubhouse members interact with external contacts (i.e., call potential employers and attend meetings with potential employers), the participants negotiated a shared stigma management strategy.

### 3.2. Research Participants

The TE meetings involved between one and six Clubhouse members and between one and three staff members. The duration of the meetings varied between 13 and 67 min (comprising a total of 794 min of interaction). Because the meetings were voluntary and open to all members of the Clubhouse, the participants varied, whereby some members attended most meetings, and some participated only once. Staff members were trained in social work and their prior work experience varied from approximately six months to several years.

### 3.3. Research Ethics

The study was conducted in accordance with the Declaration of Helsinki, and research ethics approval was obtained from the Southern Finland Clubhouse Association. The research permits were given by the board of directors of the relevant Clubhouse. Informed, written consent was obtained from all participants, and they were advised that they could withdraw their consent at any point during the study. The anonymity of the participants has been carefully ensured by altering their names and other identifying details.

### 3.4. Methodological Approach

The analysis follows the theoretical and methodological principles of discursive social psychology. According to these principles, discourse is (a) action oriented, (b) situation specific and (c) constructed and constructive [21,22]. First, in discursive social psychology, discourse is not a passageway to individuals’ thoughts and emotions but a resource for performing action [21]. Our analyses of the staff members’ discourse demonstrate how they construct stigma management strategies, but not how they ‘truly’ feel about these strategies. In line with Vesala and Knuuttila [28], non-communication, as well as self-disclosure, are functional communicative acts that can serve various interests. In the analysis, we studied how the functionality of self-disclosure and non-communication was constructed within discourse.

Second, discursive psychology recognises discursive action as being situated in sequences, argumentation and institutions. As discourse is situated in conversational sequences, discursive social psychology draws analytical tools from conversation analysis [21]. Conversation analysis reveals interaction practices through detailed microanalyses of naturally occurring interaction situations [35]. However, discursive action is also situated in argumentation and in an institutional context. As suggested by Billig [36], relevant descriptions are commonly organized to counter potential alternatives. An explicit recommendation to non-communicate a stigmatizing characteristic is, at the same time, an implicit recommendation to refrain from self-disclosure. The Clubhouse TE programme is, naturally, a highly specific institutional context. In the analysis, we investigated how self-disclosure and non-communication were constructed in conversational sequences but also in the argumentative and social reality of the Clubhouse and the TE Programme.

Finally, discursive social psychology views discourse as being constructed through resources such as categories, common places and metaphors, and as constructive in the sense that discourse allows us to construct versions of events, actions and structures [21]. Discourse constructs versions of self-disclosure and non-communication as stigma management strategies, but these versions build on numerous interaction practices such as categorization, which divides people into ‘normal’ and ‘stigmatized’ groups. Evaluative practices are a common part of construction processes [37]. Discursive social psychology highlights that in argumentation, the objects of evaluation, in this case self-disclosure and non-communication, do not remain fixed and unambiguous [38]. Rather, the two strategies can be constructed in divergent ways, depending on whether they are evaluated positively or negatively. In the analysis, we study how self-disclosure and non-communication, as stigma management strategies, are constructed when they are evaluated positively and/or negatively.

### 3.5. Analysis

We began our analytical process by watching and listening to the original video-recordings. During the first stage of the analysis, we identified all the sequences in which the staff members and Clubhouse members talked about contacting potential employers [22]. This collection consisted of 28 sequences. In these sequences, the staff members instructed Clubhouse members to remain calm and well informed about the principles of TE. Staff members also explained how an information letter must be sent before the call, and how the call must pursue an opportunity for a meeting. In the second stage of the analysis, we analysed two sequences in which information control in terms of the members’ mental illness was discussed. We investigated how self-disclosure and non-communication were constructed, evaluated and negotiated in these sequences [22]. The excerpts presented in the next section were translated from Finnish to English by the fourth author of this article. For the purpose of transparency and validity, we present the original data excerpt transcriptions in Finnish alongside their translations into English [39]. Transcription symbols used in the excerpts are presented in the Appendix A.

## 4. Results

### 4.1. Advocating Non-Communication

When staff members discussed making first contact with potential employers, they emphasized the importance of non-communicating Clubhouse members’ mental illnesses. Excerpt 1 presented in Table 1 begins with staff member 1’s, that is SM1’s, description of his own non-communication.

At the beginning of Excerpt 1a, staff member SM1 highlights how the Clubhouse organization has been able to create hundreds of job contracts with employers (lines 1–3). He continues by noting that although he has contacted potential employers, he has tried to non-communicate the fact that he is looking for jobs for people who are recovering from mental illnesses (lines 3–6, 11–12). The pauses and disconnections in his speech indicate hesitation (lines 3–5). In the middle of the staff member’s speech, a Clubhouse member verifies the strategy of non-communication (‘yeah, it is not always worth saying’, line 8). The staff member further explains the benefits of the non-communication strategy, that non-communication is necessary because so many potential employers are prejudiced.

The discussion continues in Excerpt 1b, presented in Table 2. Another staff member, SM2, aligns with SM1 and recommends non-communicating mental illnesses.

Staff member SM2 begins her speech by elucidating how potential employers often directly ask what the Clubhouse is (lines 1–2). SM2 then aligns with SM1 and advocates non-communication as a strategy even when employers directly inquire about the nature of the Clubhouse organization. According to SM2, mental illnesses do not need to be disclosed because the Clubhouse organization is for everyone. Besides mental health rehabilitees, the Clubhouse serves ‘normal’ unemployed people who just want to get out of the house (lines 2–6). However, as Clubhouses are meant to support people living with mental illness [40], the non-communication in SM2’s account starts to resemble the strategy of intentionally providing false information, commonly recognised as lying.

Staff member SM1 clearly disputes SM2’s formulation of non-communication (‘I didn’t say it exactly like that’, line 8). As SM1 disputes her explanation, SM2 offers another account. Although originally the Clubhouse organization was for people recovering from mental illnesses, nowadays Clubhouses in Finland are open to anyone who, for whatever reason, needs a place to go during the daytime (lines 13–18). A diagnosis is not needed for entry in the Clubhouse context [41]. The discussion continues in Excerpt 1c, presented in Table 3. SM2 admits that, regardless of the non-communication strategy, the stigma of mental illness will eventually emerge.

Although staff member SM2 admits that, at some point, some members’ mental illnesses ‘come out’, she does not advocate voluntary self-disclosure. Staff member SM1 continues by categorizing potential employers into two groups: those who have personal experience of mental illnesses and those who do not. According to SM1, employers who have had their own personal experience of mental illnesses usually accept information about members’ histories of mental illnesses and are more likely to provide transitional employment opportunities (lines 8–14).

SM2 aligns with SM1 and further elaborates that employers who have no personal experience with mental illnesses are prejudiced (lines 29–31). Staff member SM3 joins the conversation by impersonating a prejudiced potential employer (‘I don’t know anyone like that’, line 33). The laughter (lines 31, 34, 38) implies that employers’ prejudice is, to some degree, a delicate topic. SM2 further recounts how prejudiced employers have negative conceptions of people with mental illnesses (lines 36–38). She nevertheless discontinues her sentence and does not give a description of these conceptions, again alluding to the delicate nature of such a description (line 38). According to SM2, prejudiced employers do not understand that Clubhouse members are normal people such as mothers, fathers, students, and even highly valued professionals such as doctors (lines 39–43). This account also shows stigma management by reframing the ‘normal’ employers as negative and the stigmatized Clubhouse members as ‘normal’ [3].

In sum, the three staff members recommended non-communication as a viable interactional strategy with potential employers. The truth about members’ mental illnesses eventually ‘comes out’ but does not require active self-disclosure. Once the truth emerges, at some point, employers who have personal experience of mental illnesses are able to see Clubhouse members as normal people and thus as potential employees.

### 4.2. Preconditions of Self-Disclosure

When the staff members discussed interaction with potential employers, they posited that Clubhouse members may conceal their mental illnesses. Table 4 presents the Excerpt 2, which begins with staff member SM2’s description of a typical meeting with a potential employer.

Excerpt 2a begins with staff member SM2’s notion that when meetings are held, potential employers have no way of knowing that one of the participants is recovering from a mental illness (lines 2–4). Nevertheless, during a meeting, a potential employer may ask a question which then leads to the revelation that a participant is recovering from a mental illness (lines 4–9). SM2 describes how in such cases, employers are usually surprised (lines 9–10)—especially those employers that are prejudiced, who hold distorted conceptions of people with mental illnesses (see also Excerpt 1c in Table 3).

Staff member SM1 reinforces SM2’s comments and notes that moments of disclosure are usually functional (lines 15–17). SM2 then elaborates on her reference to prejudiced employers with a short narrative of a potential employers’ visit to one Clubhouse. In the narrative, the prejudiced employers assumed that people who are recovering from a mental illness merely nap and engage in handicrafts, but a visit to a Clubhouse made them realize that rehabilitees perform roles such as running a cafeteria and office tasks (lines 19–28).

In Excerpt 2a presented in Table 4, the staff members appear to be advocating the disclosure of information about mental illnesses. However, the positive evaluation of the disclosure depends on two conditions: (a) the disclosure only takes place when necessary and (b) the Clubhouse members do not represent stereotypical mental patients. Following Goffman [3], in the case of disclosure, people with mental illnesses must master areas that others assume to be their downfalls, such as smooth interaction and efficient work. The continuation of the discussion presented in Table 5 demonstrates the negative evaluation of voluntary self-disclosure.

After staff member SM2’s narrative of the employers’ visit (Excerpt 2a in Table 4), a Clubhouse member offers a somewhat hesitant suggestion that one could voluntarily self-disclose one’s Clubhouse membership, and thus mental illness, when calling potential employers (lines 1–4). SM2 interrupts their suggestion and disagrees (‘You don’t need to say that’, lines 6–7). Staff member SM1 agrees with SM2 and argues that it is not necessary to disclose one’s membership status (line 9).

After the direct rejection of his suggestion, the Clubhouse member explains his suggestion: self-disclosure might reduce employers’ prejudices (lines 11–13). At this point, SM2 constructs an interesting turn and argues that self-disclosure can be an acceptable strategy later in the discussion if the conditions for such self-disclosure naturally arise (lines 15–17). Following Billig [36], the implicit argument here is against voluntary self-disclosure, especially at the beginning of the discussion. The Clubhouse member aligns with SM2’s turn and uses humour as a face-saving strategy. With his humorous voice and gesture, he emphasizes that he never intended to begin the conversation with self-disclosure (lines 19–21). SM2 and SM3 validate the member’s turn with laughter and a stamping gesture, to highlight that direct self-disclosure would be an unwise strategy as it would automatically stigmatize the caller. Although the subject seems to have been settled, the Clubhouse member continues to justify his suggestion of self-disclosure. The continuation is presented in Table 6.

The Clubhouse member once more returns to his suggestion of voluntary self-disclosure and notes that employers might automatically assume that the caller is working for the Clubhouse organization (lines 1–3). Staff member SM2 admits that this is a valid assumption (lines 5–6). The Clubhouse member then continues to advocate the self-disclosure strategy by making a clear contrast with SM2’s account of a typical meeting with employers (Excerpt 2a in Table 4). Self-disclosure might reduce employers’ prejudices and thus might act as a functional stigma management strategy.

At this point, staff member SM1 interrupts the Clubhouse member and seems to object to the strategy of self-disclosure. Although it remains unclear what he means by, ‘prejudices are our own when we talk like this’ (lines 14–15), the turn seems to function as a counterargument. SM1 argues against self-disclosure by highlighting that they should all agree that the identity of the caller is not a relevant factor. What matters is that the caller is business-like and able to ‘sell’ the idea of TE to potential employers.

In sum, the three staff members constructed preconditions for self-disclosure in interaction with potential employers. Firstly, self-disclosure is not an acceptable strategy at the beginning of an interaction, but it may be acceptable later if a natural opportunity for self-disclosure arises. This may be in the form of a direct question, the answer to which requires the disclosure of the Clubhouse member’s mental illness. Secondly, self-disclosure must be preceded by interactions during which the Clubhouse member does not present stereotypes of mental illness. Self-disclosure is thus only an acceptable strategy after the Clubhouse member has first passed as a ‘normal’ person.

## 5. Conclusions

Self-disclosure and non-communication are both dilemmatic stigma management strategies. In the TE context, the disclosure of one’s history of mental illness may lead to stigmatization and discrimination. At the same time, non-communication of one’s history of mental illness may lead to accusations of lies and deception. In this article, we investigated the ways in which self-disclosure and non-communication, as stigma management strategies, were evaluated and constructed, or talked into being during staff members’ and Clubhouse members’ interaction.

The analysed data consisted of video-recordings of group meetings in which staff members and Clubhouse members discussed potential employers and aspired to establish further contact between employers and the Clubhouse organization. In the group meetings, the staff members recommended a strategy of non-communication. When contacting a potential employer, it is always best to non-communicate the issue of mental illness. Non-communication of mental illness is something the members are implicitly encouraged to also choose when they interact within the Clubhouse community. Weiste and colleagues [41] have demonstrated how Clubhouse staff members avoid talking about members’ mental illnesses when they topicalize illnesses to explain the interruptions and stoppages in their work histories. In the study by Weiste et al. [41], staff members disregarded their members’ explanations and normalized members’ situations as typical to all humans, thus not relating to the illnesses as such. By omitting members’ references to their mental illnesses, they implicitly taught the members not to discuss their illnesses in workplace-type environments.

It remains somewhat unclear which functions and whose interest the recommended non-communication strategy could potentially serve. If the person contacting the potential employer is a Clubhouse member, non-communication could naturally protect the member from stigmatization and discrimination. However, if the person contacting the potential employer is a staff member, non-communication could not protect individual Clubhouse members. In this case, non-communication could potentially serve the purpose of continuing communication with a person who is assumed to be prejudiced.

The staff members seemed to differentiate non-communication from lying or providing false information. If a potential employer asks a direct question about a person’s mental health, disclosure is necessary. Nevertheless, the staff members stipulated numerous preconditions for self-disclosure in such cases. They argued that in the best-case scenario, mental illness naturally ‘comes out’ at some point, without active self-disclosure. However, if self-disclosure is unavoidable, it should not take place at the beginning of an interaction; only after the Clubhouse member has first passed as a ‘normal’ person. Thus, when disclosing their illnesses, people with mental illnesses should show that they are able to satisfy the standards of normality set for those without mental illness (e.g., a Clubhouse member can be a physician, Excerpt 1c in Table 3).

From the staff members’ perspective, supporting Clubhouse members’ abilities to satisfy the cultural expectations of normality seems to be at the very heart of mental health promotion [42]. However, in terms of the effectiveness of the anti-stigma work conducted at Clubhouses, it could be important to reflect upon which perspective the concept of ‘normality’ is defined through. The standards of normality expected of an individual without a mental illness may indeed be decisively different from those expected of persons with a history of mental health problems. We suggest that instead of complying with the existing expectations of normality, genuine anti-stigma work should involve tailoring those expectations of normality with reference to mental illnesses [41].

The study presented in this article naturally has its limitations. Firstly, we analysed a relatively small data-set from one Finnish Clubhouse. For this reason, the study is not able to capture all the potential ways in which self-disclosure and non-communication are discussed in Clubhouses around Finland, Europe or throughout the world. However, this fact does not make our results any less interesting or relevant. Secondly and relatedly, the results cannot be straightforwardly generalised to other settings. It is possible that in other situations, the staff members would talk about self-disclosure and non-communication in different ways. It is also possible that staff members in other Clubhouses would talk about self-disclosure and non-communication in different ways. Generalisability in qualitative research is a widely discussed topic. While some scholars argue that results of qualitative research are inherently ungeneralisable, other scholars emphasise the possibility of theoretical generalisations and focus on the wider resonance that the results may have outside the analysed cases [43,44]. There is no reason to assume that the discussions analysed in the study would be somehow particularly exceptional and diverge from the ways stigma management can be discussed in other Clubhouses.

Finally, Goffman’s [3] conceptions of stigma and stigma management have been criticized for ignoring the larger cultural, political and economic questions about where stigma is produced, by whom and for what purpose. While the idea that a person is simply ‘unwell’ might be comforting for an individual suffering from mental health problems, it disregards the unequal distribution of distress in our society [45,46] and the broader notions of power and domination [47]. Adopting a micro-sociological approach has been considered ‘conservative’ in that it has been argued that it emphasizes individual agency in terms of people’s ability to influence or determine their own fates [47]. However, as we hope has become apparent in this paper, even if the micro-level analysis of social interaction does not aim to highlight individual agency, it may point to certain participants’ lack of agency. Whereas Tyler and Slater [47] appeal to ‘rethinking stigma as a contemporary mechanism of disenfranchisement’, a micro-level analysis of social interaction can demonstrate how such disenfranchisement may take place in practice.

## Figures and Tables

**Table 1:** Excerpt 1a.

1	SM1: Ja uskokaa tai älkää, niin satoja ja satoja	SM1: And believe it or not, we‘ve made hundreds
2	työsopimuksia on näissä vuosissa tehty suoraan	andhundreds of employment contracts directly with
3	työnantajan kanssa … Ja jotenkin mä en oo hirveesti	employers over the years … and somehow I haven’t
4	korostanu … puheessani mie-, mielenter- … mä en oo	really emphasized … in my speech me-, a mental- … I
5	puhunu mielenterveydestä hirveesti enkä … paljon	haven’t talked about mental health a lot or other things
6	muistakaan näistä, vaan mä oon yrittäny-	either, but instead I’ve tried-
7		
8	JÄSEN: [Niin, ei sitä aina kannata sanoo.]	MEMBER: [Yeah, it is not always worth saying.]
9		
10	SM1: [vähän enemmän ()()] työntekijää kun, tiiättekö. …	SM1: [a little more ()()] employee when, you know. …
11	Jotenkin sen vähän yrittäny sivuuttaa ja ohittaa sen takia, et	Somehow I’ve trying a bit to avoid and skip it because
12	se on monelle aika kova pala. Tai sanotaanko, et se ei oo se,	it’s a hard pill for many to swallow. Or let’s say that, it’s
13	joillekin se ei oo sitä, ei oo mitään kontaktia koskaan ollu	not that, for some it’s not that, they haven’t had any
14	mihinkään. Ennakkoluulot on vieläkin aikamoiset vaikkei	contact with anything like this. Prejudices are still quite
15	ikinä uskois et näin on.	strong, although you’d never believe it.

**Table 2:** Excerpt 1b (continuation).

1	SM2: Niin, tietysti ne kysyykin kyllä aika usein, että mikä tää	SM2: Yeah, of course they quite often ask what this
2	Klubitalo on. Mutta se, että onhan meidän toiminta tarkotettu	Clubhouse is. But, our activities are meant for
3	ihan periaatteessa kenelle tahansa. Siis kuka tahansa saa tulla	basically anyone. I mean, anyone is allowed to come
4	meille jos haluaa, että on paljon työttömiä työnhakijoita, jotka	here if they want to, there are many unemployed job
5	ei jaksa olla kotona, että tuntuu, että seinät kaatuu päälle.	seekers, who are tired of being at home, and feel like
6		the walls are closing in on them.
7		
8	SM1: Mä en ihan sillai sanonu, [mutta siis ajatuksena-]	SM1: I didn’t say it exactly like that, [but the idea-]
9		
10	SM2: [Ja niinku-] Niin, niin, mut siis se, että, että ei voi, mun	SM2: [And like-] Yeah, yeah, but the fact, that, that it
11	mielestä on ihan yhtä väärin sanoa se, että, että sanoa tän	can’t, I think it’s just as wrong to say that, that to say
12	olevan mielenterveyskuntoutujille tarkotettua, koska siis ei	this is for mental health rehabilitees, because like not
13	kaikilla ole välttämättä mitään. Siis vaikka tää klubitalomalli	everyone has something. Although this Clubhouse
14	on lähtenyt aikoinaan siitä, mutta eihän se Suomessa enää sitä	model began from that, it isn’t that anymore in
15	ole. On opiskelijoita, jotka … vaikka sen takia, että on lonkka	Finland. There are students who … like, because of a
16	leikattu ja ((nauraa)) ei pääse niin kun puoleen vuoteen	hip operation and ((laughing)) can’t go anywhere for
17	mihkään ja tuntuu, että seinät kaatuu päälle, niin tulee tänne.	six months and feel like the walls are closing in on
18	Tai sit niin kun, niin. Et jollakin laillahan se täytyy tietysti	them, so they come here. Or then like, yeah. Like, of
19	perustella, koska ne kysyy mitä tää klubitalotoiminta on	course we have to explain it in some way, because
20	[niinku mutta]	they ask what this Clubhouse is for [like, but]
21		
22	SM1: [Hmm, mmm.]	SM1: [Hmm, mmm]

**Table 3:** Excerpt 1c (continuation).

1	SM2: Mutta että tietysti se tulee esiin joka tapauksessa, että	SM2: But of course, it will eventually come out that
2	on myös niitä ihmisiä, joilla on ollut mielenterveyden	there are also people who have had mental health, like,
3	kanssa niin kun ongelmia tai tai näitä.	problems or, or something.
4		
5	SM1: Joskus sitä muuten vähän tutkittiin tai mietittiinkin	SM1: It was actually studied a bit or thought through
6	sillon aikoinaan, ((SM2:n nimi)) sillon, oltiinko kollegoja	at some point, ((SM2’s name)) then, were we
7	joskus aikoinaan, mutta mietittiin sitä, et saatiin selville	colleagues at that time, but we thought about it, and
8	sellasta työnantajista, että aika useesti ne työnantajat ja ne	we found out about those kinds of employers, that
9	työ-, ne työ- työhön ottajat, jotka vastas työhön otosta taikka	quite often the employers and the work, the work-,
10	jotenkin johti sitä firmaa, niin sellaset työnantajat aika	those recruiters who were in charge of recruitment or
11	useesti otti nää asiat niin kun, otti otti ihmisiä töihin, joilla	somehow led the company, that those kind of
12	oli jotain [henkilökohtasta] kokemusta asiasta.	employers quite often took these things in the way that
13		they employed people who had some [personal]
14		experience of the matter.
15		
16	JÄSEN: [Mm.]	MEMBER: [Mm.]
17		
18	SM1: Ja sit se jotenkin kävi ilmi niissä keskusteluissa, että-	SM1: And then it somehow came out in those
19		discussions, that-
20		
21	JÄSEN: Tai niin suku, suvussa on-	MEMBER: Or that the family, the family has-
22		
23	SM1: Sukulaisissa on tai jossain muissa, ja yllättävän	SM1: Relatives have or someone else, and surprisingly
24	monella niitä oli aika lähipiirissäkin. Ja se niin kun tavallaan	many had them in, like, their inner circle. And it, like,
25	herätti niissä sellasen ajatuksen, että täähän on niin kun tosi	evoked an idea in them, that this is like a really good
26	hyvä juttu. Se oli aika jännä, sitä joskus mietittiin mistähän	thing. It was quite interesting, we tried to think at some
27	se vois johtuu.	point what the reason could be.
28		
29	SM2: Ja sitten on vähemmän ennakkoluuloja semmosilla	SM2: And then there is less prejudice in those people,
30	ihmisillä, kun on sit niitä, jotka luulee, ettei kellään yhtään	whereas there are others who think that no one at all
31	((naurua)) ihmisellä oo koskaan ollut mitään.	((laughter)) has ever had anything.
32		
33	SM3: Minä en tunne yhtään sellaista ((ilmeilee ja puhuu	SM3: I don’t know anyone like that ((make faces and
34	leikkisästi)) ((naurua yhdessä))	speaks playfully)) ((laughter together))
35		
36	SM2: Niin minä en tunne ketään sellaista, niin sitten tota	SM2: Yeah, I don’t know anyone like that, and then,
37	niin niil on joku ihan omanlainen niin kun häiriintyny	like, you know, like they have their own kind of
38	käsitys siitä, ((naurua)) että mitä mimmosia niinku. Et siis	twisted perception of it ((laughter)) like what kind of,
39	mä aattelen, että ketkä on Klubitalolla käyviä ihmisiä?	like. So, I’m thinking who are the people who go to
40	Tavallisia perheen äitejä ja isiä, opiskelijoita, kaikki	Clubhouses? Normal mothers and fathers, students,
41	maailman niinkun yhteiskuntaluokat ammatit on niin kun	all possible social classes occupations there are like
42	metsureista lääkäreihin, kaikkea löytyy niinku Klubitalon	from lumberjacks to doctors, allsorts, like, among
43	ihmisistä.	Clubhouse people.

**Table 4:** Excerpt 2a.

1	SM2: Mieluummin aina niinku pareittain, niin ainahan	SM2: It’s better to always do it in pairs, so there’s always
2	siellä on niinku henkilökuntajäsen ja jäsen. Eihän se	a staff member and a member. After all, they don’t
3	koskaan tiedä … et millä mikä niinku meidän …	know … like what our … ((laughter)) background is,
4	((naurahtaa)) tausta on ketkä sinne on lähteny. Et sit jossain	who come there. And then at some point in the
5	kohtaa keskustelussa voi tulla ilmi että, työnantaja on jotain	conversation it may come out that, the employer has
6	kysyny ja sitte on käyny ilmi että okei et mä oon mä oon	asked something and then it turns out that okay I’m, I’m
7	palkatussa työsuhteessa siellä Klubitalolla ja tää toinen	an employee at the Clubhouse and this other person is
8	henkilö on jäsenenä siellä Klubitalolla ja sitte ollaan että	a member of the Clubhouse and then they’re like ahaa
9	ahaa ((esittää yllättynyttä ilmettä)). Et se on tullu	((acts surprised)). So, it’s come as a surprise. That
10	yllätyksenä. Että varsinki semmosilla jotka … niinku …	especially for those who … like … truly their own
11	tosiaan oma mielikuvitus on kehittänyt omia juttuja et aijaa	imagination has developed its own stories and then
12	((yllättyneellä äänellä)) tässä on ihan niinku ihan niinku	they’re like is that so? ((with a surprised voice)) the
13	normaaleja ihmisiä tässä nyt ((naurahtaa))	people here now are just, like, just normal ((laughter))
14		
15	SM1: Niin ku ne ennakkoluulot on semmosia että ne. Mutta	SM1: Yeah, prejudices are such that they tend to. But
16	se seki monta kertaa [laukasee tilannetta hyvään suuntaan].	that, that also often [steers the situation in a good
17		direction.]
18		
19	SM2: [Et se on vielä. Niin.] O. Ja sitte joskus tota mitä saatiin	SM2: [Like it still is. Yeah.] It is. And then, Erm, once we
20	((paikan nimi))) Klubitalolle sillai että työnantajat tulikin	got ((name of the place)) to the Clubhouse so that
21	sinne Klubitalolle … niin ne oli ihan sillee et ei vitsi että tää	employers came there to the Clubhouse… so they were
22	on aivan erilaista kuin mitä he on niin kun ajatellu. Ja sit ne	just, like, no way, that this is totally different to what
23	on ollu nimenomaan positiivisesti yllättyneitä. Ja sit ne on	they had, like, expected. And they‘ve been positively
24	aatellu että että no täällähän tehään oikeesti niinku asioita,	surprised. And then they’ve thought that people really
25	et tääl ei tosiaan virkata ja kudota poppanaa ja joku käy	do things here, people are not crocheting and or
26	torkkumassa tossa. Vaan et täällä tehdään oikeesti niinkun	weaving tablecloths here and someone snoozing there.
27	asioita. Että pyöritetään ravintolaa ja tehdään kaikki niinku	But, like, they really, like, do things here. That we run a
28	toimiston … työt että.	cafeteria and do all the kind of office … jobs so.

**Table 5:** Excerpt 2b (continuation).

1	JÄSEN: Ehkä tää. No siis mä mietin että onks siinä mitään	MEMBER: Maybe this. Well, I was wondering if there
2	järkeä että ku soittaa niin siinä vaiheessa että … tai no niin	was any point in, like when you call, at that point that …
3	no joo … tarkotan että tuo sitä ilmi että et se soittaja on	or well yeah ok … I mean that you reveal that the caller
4	niinku … ei oo työntekijä vaan se on-	is like … is not an employee but a-
5		
6	SM2: Ei sitä tartte sanoo, [vaan että Klubitalolta soittaa.]	SM2: You don’t need to say that, [just that you’re calling
7		from a Clubhouse.]
8		
9	SM1: [Ei sitä tartte sanoo.]	SM1: [You don’t need to say it.]
10		
11	JÄSEN: [Eiku mä aattelin vaan] niinku että auttaako se	MEMBER: [No, I was just thinking] like would it help
12	osaltaan niihin ennakkoluuloihin. Tavallaan jos se niinku	with the prejudice. Like if the person realized that the
13	huomaa että puhelimessa onki niinku-	caller was, like-
14		
15	SM2: No voi se olla jos sitten niinku jossain kohtaa	SM2: Well, it could be that sometime later on in the
16	pidemmällä keskustelu viriää ja tulee esiin jotain [missä voi	conversation something may come up [and then you
17	ittensä esittää]	can introduce yourself]
18		
19	JÄSEN: [Niin emmä heti alussa sillee] että että jäsennumero	MEMBER: [Yeah, I don’t, not right at the beginning]
20	[seiskytkaks täällä] ((vakavalla, virallisella äänellä))	like like member number [seventy-two here] ((in a
21		serious, official voice))
22		
23	SM2: [((nauraa))] Niin just toi oliski just hyvä lähtökohta	SM2: [((laughs))] Yeah that would be a great starting
24	((nauraa)) lähestymistapa.	point ((laughs)) approach.
25		
26	SM3: ((tekee eleen ja äänähtää ikään kuin leimaisi käteensä))	SM3: ((makes a gesture and sound as if stamping own
27		hand))

**Table 6:** Excerpt 2c (continuation).

1	JÄSEN: Mut siis se että silleen justiinsa, ku mä aattelin vaan	MEMBER: But like, just like, I was just thinking that if,
2	et jos jos niinku työnantajalla on niinku se kuvitelma, että	if like, the employer gets the impression that the caller
3	sieltä soittaa et et-	is, is -
4		
5	SM2: Niin todennäkösesti, voisin olettaa että ne vois ajatella	SM2: Yeah most likely, I could imagine they might think
6	niin	so
7		
8	JÄSEN: Että se on työntekijä joka soittaa. Mut sitte jos ne	MEMBER: That it’s the employee who is calling. But
9	niinku käsittää et se on jäsen joka soittaa, niin aijaa se oliki	then if they, like understand that it is a member calling,
10	ihan niinku et ((yllättyneellä äänellä)) se ei- tai niinku että	then oh wow it wasn’t, like ((in a surprised voice)) it’s
11	niin en mä tiiä	not- or like that yeah, I don’t know
12		
13	SM1: Nyt meidän täytyy aina muistaa et nyt ne	SM1: Now we always have to remember that these
14	ennakkoluulot on meissä ittessämme ku me puhutaan	prejudices are our own when we talk like this ((SM2 and
15	tämmöstä ((SM2 ja SM3 nyökyttelevät)). Se on niille ihan eri	SM3 nod)). It’s a whole different world for them. So,
16	maailmaa. Eli tota noin niin … niin tota …	well err… so erm…
17		
18	JÄSEN: Mutta joskus ennakkoluulot on tietenkin pohjassa ()	MEMBER: But sometimes prejudices are, of course,
19	()	underneath it all ()()
20		
21	SM1: Se on hirveen tärkeetä olla asiallinen siinä puhelimessa	SM1: It’s terribly important to be business-like on the
22	ja tietää mistä puhuu, Et sillä ei oo mitään, meijän kannattaa	phone and to know what you’re talking about, that it
23	heittää se ajatus kokonaan pois että kuka soittaa, on kuka, sil	doesn’t, we should completely throw away the idea that
24	ei oo mitään väliä. Mut se et ku me myydään sitä hommaa	it matters who’s calling, who it is, it doesn’t matter. But
25		
26	niin pitää jotenkin niinku pystyä tarttuun hetkessä sit niihin	because we’re selling this, somehow we have to be able
	juttuihin mitä siinä puhuu.	to immediately deal with the things we’re talking about

## Data Availability

The original video recordings analysed during the current study are not publicly available due to privacy restrictions, but anonymized transcripts are available from the corresponding author on reasonable request.

## Appendix A

Transcription symbols

[ ]	Overlapping talk
…	A pause
word-	Disconnection in the sentence
( )	Transcriber could not hear what was said
((word))	Transcriber’s comments or description of phenomena
